# In-house coordination project for organ and tissue procurement: social responsibility and promising results**^1^**

**DOI:** 10.1590/1518-8345.0841.2773

**Published:** 2016-07-25

**Authors:** Vanessa Silva e Silva, Luciana Carvalho Moura, Luciana Ribeiro Martins, Roberta Cristina Cardoso dos Santos, Janine Schirmer, Bartira de Aguiar Roza

**Affiliations:** 2 Doctoral Student, Escola Paulista de Enfermagem, Universidade Federal de São Paulo, São Paulo, SP, Brazil, and Queen´s University at Kingston, Kingston, ON, Canada.; 3 RN, Hospital Israelita Albert Einstein, São Paulo, SP, Brazil.; 4 RN, Specialist in Organ Donation, Hospital Israelita Albert Einstein, São Paulo, SP, Brazil.; 5 PhD, Full Professor, Escola Paulista de Enfermagem, Universidade Federal de São Paulo, São Paulo, SP, Brazil.; 6 PhD, Adjunct Professor, Escola Paulista de Enfermagem, Universidade Federal de São Paulo, São Paulo, SP, Brazil.

**Keywords:** Healthcare Financing, Tissue and Organ Harvesting, Nursing, Tissue and Organ Procurement

## Abstract

**Objectives:**

to report the results of evaluation regarding changes in the number of potential donor referrals, actual donors, and conversion rates after the implementation of an in-house organ and tissue donation for transplantation coordination project.

**Methods:**

epidemiological study, both retrospective and transversal, was performed with organ donation data from the Secretariat of Health for the State and the in-house organ donation coordination project of a beneficent hospital. The data was compared using nonparametric statistical Mann-Whitney test, and the Student's t-test, considering a significance level of 5% (p <0.05).

**Results:**

there were statistically significant differences (p < 0.05), before and after the implementation of the project on the number of potential donor notification/month (3.05 - 4.7 ), number of actual donor/month (0.78 to 1.60) and rate of conversion ( 24.7 to 34.8 %). The hospitals 1, 2, 7 and 8 had significant results in potential donor, actual donor or conversion rate.

**Conclusion:**

the presence of an in-house coordinator is promising and beneficial, the specialist is important to change the indicators of efficiency, which consequently reduces the waiting lists for organ transplants.

## Introduction

In Brazil, the organ and tissue donation and transplantation's national system was implemented in 1997 by the law No. 9,434 and the decree No. 2,268^1^ ^-^ ^2^. After this federal regulation some states started to create specific rules and resolutions to deal with this topic locally. In São Paulo, the local organizational and operational structure of the organ donation and transplant system was determined by the resolution SS No. 103/1997^3^. Therefore, it was established the organ donation model adopted there, which was based on the North-American's model of Organ Procurement Organization.

The Organ Procurement Organization is based on regionalized professional support for hospitals to administrate the entire organ donation process. This support is provided by external expert nurses and/or physicians. However, it is known that in-house committees for organ donation and transplantation allow a better organization of the organ donation process. The Spanish model, which preaches the presence of an organ and tissue donor coordinator (OTDC) in these in-house committees for organ donation, facilitates the early identification of potential organ donors, and provides an adequate family support. This structure also allows better communication amongst all the structures of the organ donation and transplantation system, consequently improving quantitatively and qualitatively the organ donation^4^ ^-^ ^5^. 

Therefore, the state of São Paulo made some changes in its organ donation system and adopted some particularities of the Spanish model. From this point, São Paulo's organ donation system became a blend of the north-American's and the Spanish's models. This new model incorporated the in-house committees for organ donation. In Brazil, the in-house committees are classified by the number of deaths per year in their hospitals. The type I refer from 0 to 200, the type II above 200 and type III above 1,000 deaths per year^6^. 

This committee is composed by a multidisciplinary team and usually has a nurse as coordinator^7^.The activity of nurses in the organ donation and transplantation field is regulated by the Conselho Federal de Enfermagem (nursing federal council - COFEN) resolution No. 292/2004. This resolution describes their responsibility to manage the whole process of organ donation: from the identification of the potential organ donor to the release of the body for the family to the funeral ceremonies^8^. Although the nurses possess legal support to work with organ donation, in Brazil the majority of nurses do not perform this activity exclusively. These nurses usually work in acute care and along with this position they perform the organ donation activities. This prevents the hospital from achieving their potential for organ donation, since the nurses are not exclusive for managing the whole process.

In order to address this problem, some initiatives arose in the state of São Paulo. One of them was implemented in 2008 as a partnership among one beneficent hospital in São Paulo, the National Transplant System, and the Secretariat of Health for the State of São Paulo. This beneficent hospital has developed a project of in-house organ and tissue donation for transplantation coordination for public hospitals. This was a pioneer project that hired a specialist nurse to act in public hospitals as an OTDC, at no cost to these hospitals. The objective of this project was to increase the number of organ and tissue donors and, consequently, reduce patients' waiting list for transplants. To understand the effectivity of this strategy's results, the project was evaluated after five years.

Therefore, the aim of this study was to report the results of evaluation regarding changes in the number of potential donor referrals, actual donors, and conversion rates after the implementation of an in-house organ and tissue donation for transplantation coordination project.

## Methods

This epidemiological study, both retrospective and cross-sectional, was performed with organ donation data from the Secretariat of Health for the State of São Paulo and the in-house organ donation coordination project of a beneficent hospital, from 2003 to 2012. This research was approved by the ethics committee of Federal University of São Paulo, Brazil.

The study population was made up based on the potential donor referrals and number of actual donors, from the state of São Paulo, with a sample before and after the nursing specialist's arrival at nine public hospitals. The arrival of the specialist nurses was considered as the intervention in the nine hospitals. The specialist nurses' work was to identify potential organ donors, improve the organ donor maintenance, and provide family support. Furthermore, these nurses had an educative role with the intensive care team in order to teach them about the importance of early potential donor identification and prevent donor loss due to poor maintenance. The selection criterion of the hospitals was to have at least six months experience with an in-house coordinator hired by the in-hospital coordination for organ donation project at the beneficent hospital. All nine hospitals provide high complexity care, including treatment for cases of trauma, neurology or neurosurgery.

For the purpose of patterning the terms within the scientific nomenclature, the terms "potential donor referrals" and "number of actual donors" will be replaced herein by: *potential donor after brain death* (PDBD is a person whose clinical condition is suspected to fulfill brain death criteria); and *actual donor after brain death* (ADBD is a consented eligible donor in whom an operative incision was made with the intent of organ recovery for the purpose of transplantation)^9^. 

The data was collected during a single occasion and it was separated into two groups: one before the beginning of the project (September 2003 to September 2011) and one after (May 2008 to December 2012), with an equal number of months being evaluated before and after the project was implemented at each hospital.

The collection instrument was developed using a spreadsheet (Microsoft Excel(r)) with variables related to the number of PDBD and ADBD at the nine hospitals in which the project was hosted, numbering the hospitals from one to nine to ensure anonymity. The numbers of PDBD and ADBD were transcribed from the organ donation project database to the spreadsheet and were validated with official information from the database of the Secretariat of health of the state of São Paulo. 

The instrument was built based on current literature and on the researcher's experience. The validation of layout and content of the instrument was conducted by the researchers members of the Study group in Organ Donation and Transplantation - Federal University of São Paulo (GEDOTT - Unifesp) in a single meeting, being obeyed all suggestions made by the group members. 

The data was statistically analyzed using the s*tatistical package for the social sciences* (SPSS^(r))^ software. For comparisons were used the nonparametric statistical Mann-Whitney test, with significance level of 5% (p <0.05) and the Student's t-test, considering a significance level of 5% (p <0.05). The interest in this analysis was to compare the number of PDBD and ADBD before and after the nursing specialist's arrival at the nine public hospitals. For both periods it was used as a landmark the month which the nurse began the work in the hospitals studied. The analysis was performed in three stages: first the comparison of the whole data for the differences of PDBD and ADBD numbers before and after the presence of the nurse specialist; second an individual analysis of the hospitals to identify statistical significant changes in these variables and; last the verification of conversion rates, which is measured by dividing the numbers of ADBD by PDBD. 

The data collected was also compared with the performance indicators described in the ordinance No. 1.262/2006: occurrence of brain death estimated between 10-14% from all hospital deaths; and achievement at least of 30% of the effectiveness in donation from the number PDBD referred to the transplant central (conversion rates)^10^.

The average of notifications of PDBD per year number was inferred by multiplying the number of PDBD per twelve months for each hospital for both periods, before and after the project. 

## Results

There were a total of 1,080 notifications of PDBD and 364 ADBD among the nine hospitals, during the period from 2008 to 2012. The number of PDBD notification presented a statistically significant difference (p <0.05), indicating increased values ​​of PDBD notification after the presence of the nurse specialist (Table 1). Before the nurse there were 3.05 PDBD notifications/month, which increased to 4.74 in the period right after the implementation of the project (Table 1). 

**Table 1:** Number of notification of potential donor after brain death and actual donor after brain death before and after the implementation of the in-house organ donation coordination project. São Paulo, SP, Brazil, 2003 to 2012

Type of Donor	Variable	Intervention		p value
		Before the intervention	After the intervention	
PDBD*	mean/month	3.05	4.74	<0.001
	Median	2.00	4.00	
	Standard deviation	2.78	3.70	
	Minimum	0	0	
	Maximum	14	17	
ADBD†	mean/month	0.78	1.60	<0.001
	Median	1.00	1.00	
	Standard deviation	0.96	1.51	
	Minimum	0	0	
	Maximum	5	8

PDBD*: Potential donor after brain death; ADBD†: Actual donor after brain death

Analyzing the hospitals separately for number of PDBD notification, we can observe the statistical significance (p <0.05) in three of the nine hospitals: *hospital 1* from 4.88 to 8.39 per month, *hospital 2* from 1.97 to 7.05 per month and *hospital* 8 from 0.58 to 1.54 per month. The results also show a difference (p <0.05) in the numbers after the presence of the specialist, the ADBD per month increased from 0.78 to 1.60 (Table 1). For the number of ADBD, it was observed statistical significant difference in three hospitals: *hospital 1* from 1.02 to 2.02 per month, *hospital 2* from 0.30 to 2.59 per month and *hospital 7* from 1.38 to 2.42 per month.

The conversion rate in general presented a difference (p<0.05) ranging from 24.7% to 34.8% after the presence of the nurse specialist (Table 2). Analyzing the hospitals separately, we observe that the *hospitals 2* and *4* demonstrated statistical significance in conversion rate, indicating a higher conversion rate after the presence of a specialist, from 11.1% to 35.1% and from 27.3% to 46.1%, respectively.

**Table 2:** Difference of the conversion rate of notification of potential donor after brain death to actual donor after brain death before and after the implementation of in-house organ donation coordination project. São Paulo, SP, Brazil, 2003 to 2012

Indicator	Variable	Intervention		p value
		Before the intervention	After the intervention	
Conversion rate	mean/month	24.7%	34.8%	<0.001
	Median	16.7%	33.3%	
	Standard deviation	29.5%	29.6%	
	Minimum	0.0%	0.0%	
	Maximum	100.0%	100.0%

The occurrence of brain death was estimated in 20 brain deaths per year in hospitals with in-hospital committee type II, and 100 brain deaths per year in the type III. The conversion rates between hospitals ranged from 25.7% to 48.6%, with an average of 24.7% before and 34.8% after the project. The average number of notifications of PDBD per year was inferred for each hospital displayed on Table 3:

**Table 3:** Average number of PDBD notifications per month and per year before and after the implementation of in-house organ donation coordination project. São Paulo, SP, Brazil, 2003 to 2012

Hospital	Type of In-house committee Per month		Before the Project		After the Project	
			Per year	Per month	Per year	
1	III		4.88	58.56	8.39	100.68
2	III		1.97	23.64	7.05	84.6
3	III		1.5	18	2.13	25.56
4	III		3.65	43.8	2.59	31.8
5	III		1.25	15	1.95	23.4
6	III		2.05	24.6	2.15	25.8
7	II		4.92	59.04	5.08	60.96
8	II		0.58	6.96	1.54	18.48
9	II		1.47	17.64	1.27	15.24

## Discussion

The results presented in this article are positive and they show the effectiveness of the project in terms of number of potential organ donor's referrals and effective donors. Some hospitals did not reach the estimated occurrence of brain death determined by the ordinance No. 1,262/2006, due to the fact that these hospitals had the project for a short time or because the nurse quit the job.

In the other hand, hospitals with more time participating in the project, i.e. *hospitals 1* and *2*, had rates of PDBD/year significantly closer to the estimated values for the occurrence of brain death described on the ordinance No. 1.262/2006^10^.

There is one hospital (*hospital* 7) that stood from the others in numbers of PDBD, because it was strategically located next to a highway, attending high complex trauma and being a reference to health care in the area. Furthermore, the specialist nurse in charge for the project in this hospital performed an important educational role within the health care professionals and the community. The remaining hospitals had results close to the values ​​for the occurrence of brain deaths estimated by the ordinance mentioned above.

Furthermore, this study demonstrated that the majority of the hospitals evaluated succeeded in achieving the goal recommended by the ordinance No. 1.262/2006 concerning conversion rate. It was observed that the rates of the hospitals ranged from 25.7% to 48.6%, with an average of 24.7% before and 34.8% after the project. Although the conversion rates were higher than the recommended by the ordinance No. 1,262/2006, they are still low compared to conversion rates of countries like the United State of America, for example, which presented 73.4% conversion of potential donors into effective donors in the year of 2011^11^. 

We can argue that the proper functioning of an in-house committee is pretty important for the improvement of numbers of effective donors^12^. By the results presented we can understand that the in-house committees for organ and tissue donation for transplantation improve the processes involved in organ donation. These committees ensure better quality and quantity of organs supplied to the country's public transplant system ^13^ ^-^ ^15^. What can be seen by the statistical significant difference in the number of brain death notifications after the arrival of the specialist nurse in the hospitals studied and also in the effective donors generated for the transplant state system. The consequence of these numbers would reduce the waiting lists for transplantation, through increasing the possibility of equity, which is one of the guiding principles of the Brazilian public health system. After an organ transplant the patient will be able to return to the labor market, since many rely on state benefits, such as pensions, to subsist during the pre-transplantation phase. Beyond that, studies indicate that indirect costs of a patient in the transplant waiting list are higher than the organ transplant procedure^16^ ^-^ ^17^.

These increasing results presented in numbers of brain death notification and effective donors can be explained by the educational and structural interventions applied by the specialist nurses, such as training the hospital staff; introduction institutional records specific to manage the organ donation process (specific forms); and implementation of standardized evidence based medical prescription for maintaining the potential organ donor.

These positive results of the interventions applied by the specialist nurse are clear when we look at the results of *hospitals 2* and *7.* We can see that the potential donors before unidentified, started being notified after the work of the specialist nurse, evidencing the real potential for organ donation of these hospitals according to the type of committee (type III). Other reasons that influence positively the results are the interference that facilities related to organ and tissue donation can have in the organ donation committees. For example, the results presented for the *hospital 1* could have been positively influenced by the work of an eye bank, which made the local population to get used to the organ and tissue donation, and also because the hospital is reference for taking care of trauma and neurological problems.

Apart from this project, we can notice that the presence of the OTDC with workload and exclusive payment to perform activities of organ donation is a reality in few parts of Brazil. The problem is that Brazil does not have formal regulations to support the payment of specialist nurses to work as OTDC. This makes us inquire the urgent need of a formal professionalization of in-house committees with payment and exclusive workload for the coordinator as a result of developments in the area in our country.

The main strength of this study was the long term analysis performed amongst the hospitals. However, this was the unique initiative for organ donation of this type in the state of São Paulo at the time of the study. There was no control group, constituting a limitation of the study. Future studies must be multicenter with a larger number of hospitals and comparing the data long term. Even so, this study is pioneer in Brazil and demonstrated the importance of the specialist nurse as an OTDC to improve the organ donation process.

## Conclusion

The report presented in this study showed that the presence of an organ tissue donor coordinator (specialist nurse) is beneficial and socially promising. We observed a clear improvement in the number of brain death notifications in the period after the implementation of the project. Even so, this represents a daily challenge for in-house coordinators, since the diagnosis of brain death is a medical expertise and it is known to still have many cases of underreporting.

This simple fact of increasing brain death notifications create greater possibilities for effective donors, ensuring both the right of diagnosis of brain death to the patient and the right to decision of the families of the deceased. In this scenario, the OTDC improves the family embracement because there is a unique team for this work. 

Finally, a more careful government attention on the policies will be necessary, especially for those that rule the organ donation and transplantation processes. Furthermore, funding alternatives for payment of the costs with this important professional need to be found.

## References

[1] (1997). Law No. 9434 February 4th, 1997 (BR). Provides for the removal of organs,
tissues and body parts for transplantation and treatment and other
measures.

[2] (1997). Decree No. 2.268 June 30th, 1997 (BR). Regulates Law No. 9,434, of February
4, 1997, which provides for the removal of organs, tissues and body parts for
transplantation and treatment, and other measures.

[3] Resolution SS - 103.August 1th.1997 (1997). Provides for the organizational and operational structure of the State System
Transplant São Paulo.

[4] Salim A, Berry C, Ley EJ, Schulman D, Desai C, Navarro S (2011). In-house coordinator programs improve conversion rates for organ
donation. J Trauma.

[5] Shafer TJ, Ehrle RN, Davis KD, Duran RE, Holtzman SM, Buren CTV (2004). Increasing organ recovery from level I trauma centers the in-house
coordinator intervention. Progress Transplant.

[6] (2009). Ordinance No. 2600, October 21th, 2009 (BR). Approves the Technical
Regulation of the National Transplant System.

[7] Knihs NS, Schirmer J, Roza BA (2011). Adaptación del Modelo Español de Gestión en Transplante para la Mejora
en la Negativa Familiar y Mantenimiento del Donante Potencial. Texto Contexto Enferm.

[8] Nursing Federal Council (2004). No. 292/2004. Regulates the activities of the Nurse Recruitment and
Transplantation of Organs and Tissues.

[9] Domínguez-Gil B, Delmonico FL, Shaheen FAM, Matesanz R, O'Connor K, Minina M (2011). The critical pathway for deceased donation reportable uniformity in
the approach to deceased donation. Transplant Int.

[10] (2006). Ordinance GM No. 1.262, June 16th, 2006. Approves the Technical Regulation to
establish the powers, duties and efficiency indicators and the potential of organ
and tissue donation related to In-house committees for organ donation and
transplantation.

[11] U. S. Department of Health & Human Services (UNOS) (2011). Annual Data Report.

[12] Silva OC, Souza FF, Nejo P (2011). Donation Organ Transplants in Brazil what's missing? What can be
done?. ABCD Arq Bras Cir Dig.

[13] Arcanjo RA, Oliveira LC, Silva DD (2013). Reflections about the Intra-hospital Commission on Organ and Tissue
Donation for Transplant. Rev Bioética.

[14] Elizalde J, Lorente M (2006). Coordinación y Donación. An Sist Sanit Navar.

[15] Moraes EL, Massarollo MCKB (2008). Family refusal to donate organs and tissue for transplantation Rev.
Latino-Am. Enfermagem.

[16] Caplan AL (1983). Organ Transplants The Cost of Success. The Hastings Center Report.

[17] Marinho A, Cardoso SS, Almeida VV (2010). Geographic disparities in organ transplantation in
Brazil. Cad Saúde Pública.

